# Chronic Serotonergic Overstimulation Mimicking Panic Attacks in a Patient with Parkinson's Disease Receiving Additional Antidepressant Treatment with Moclobemide

**DOI:** 10.1155/2021/8868023

**Published:** 2021-03-01

**Authors:** Marc Praetner, Timo Schiele, Lukas Werle, Janina Kuffer, Sandra Nischwitz, Martin E. Keck, Stefan Kloiber

**Affiliations:** ^1^Walter Brendel Center of Experimental Medicine, Ludwig-Maximilians-Universität München, Marchioninistr. 15, 81377 Munich, Germany; ^2^Psychosomatische Klinik Kloster Dießen GmbH & Co. KG, Klosterhof 20, 86911 Dießen am Ammersee, Germany; ^3^Benedictus Krankenhaus Tutzing Bahnhofstraße 5, 82327 Tutzing, Germany; ^4^Algesiologikum GmbH, Heßstr. 22, 80799 Munich, Germany; ^5^Max Planck Institute of Psychiatry, Kraepelinstrasse 2-10, 80804 Munich, Germany; ^6^Kliniken Schmieder 78260 Gailingen am Hochrhein, Germany; ^7^Department of Psychiatry, University of Toronto, Canada; ^8^Campbell Family Mental Health Research Institute, Centre for Addiction and Mental Health, 100 Stokes Street, Toronto, ON M6J1H4, Canada

## Abstract

**Background:**

The pharmacological treatment options of Parkinson's disease (PD) have considerably evolved during the last decades. However, therapeutic regimes are complicated due to individual differences in disease progression as well as the occurrence of complex nonmotor impairments such as mood and anxiety disorders. Antidepressants in particular are commonly prescribed for the treatment of depressive symptoms and anxiety in PD. *Case Presentation*. In this case report, we describe a case of a 62-year-old female patient with PD and history of depressive symptoms for which she had been treated with moclobemide concurrent with anti-Parkinson medications pramipexole, rasagiline, and L-DOPA+benserazide retard. An increase in the dosage of moclobemide 12 months prior to admission progressively led to serotonergic overstimulation and psychovegetative exacerbations mimicking the clinical picture of an anxiety spectrum disorder. After moclobemide and rasagiline were discontinued based on the hypothesis of serotonergic overstimulation, the patient's psychovegetative symptoms subsided.

**Conclusions:**

The specific pharmacological regime in this case probably caused drug-drug interactions resulting in a plethora of psychovegetative symptoms. Likely due to the delayed onset of adverse effects, physicians had difficulties in determining the pharmacologically induced serotonin toxicity. This case report emphasizes the complexity of pharmacological treatments and the importance of drug-drug interaction awareness in the treatment of PD patients with complicating nonmotor dysfunctions such as depression.

## 1. Background

Parkinson's disease (PD) is a neurodegenerative disorder characterized by the progressive occurrence of typical motor dysfunctions such as resting tremor, rigidity, bradykinesia, and postural instability as well as concurrent nonmotor dysfunctions such as hyposomnia, REM (rapid eye movement) sleep behavior disorder, depression, autonomic dysregulation, cognitive impairments, psychosis, and anxiety disorders [1]. This clinical complexity of PD is mirrored by the current neuropathological understanding of PD. Subsequent disruption of nigrostriatal dopaminergic neurotransmission through degeneration of mesencephalic neurons located in the substantia nigra is accompanied by neurodegenerative processes in other brain areas leading to complex disturbances of neurobiological systems beyond the dopaminergic system including serotonergic and noradrenergic pathways [2]. Notably, dopaminergic dysfunction and serotonergic neuropathology of the basolateral amygdala were associated with anxiety in a rat model of PD and responsive to treatment with levodopa (L-DOPA) [3]. In the latest version of the Diagnostic and Statistical Manual of Mental Disorders, fifth edition (DSM-5), anxiety spectrum disorders consist of 12 diagnostic entities [4]. A panic attack is defined as a “discrete period of intense fear or discomfort” where symptoms such as palpitations, sweating, trembling or shaking, shortness of breath, chest pain or discomfort, nausea, dizziness, derealisation or depersonalisation, and fear of dying develop abruptly and can reach their peak within minutes [5]. In a recent cross-sectional study, Rai and colleagues investigated the prevalence of neuropsychiatric disorders, focussing on depression, anxiety, and psychosis in patients with PD. The results indicate depression to be the most common neuropsychiatric comorbidity with a prevalence of 43.7%; anxiety was found in 35.7% of patients with PD [6]. A high prevalence of anxiety disorders in PD was confirmed in other studies [7–9] describing a heterogeneous clinical picture with both sustained and episodic anxiety [8]. However, specific treatment options for anxiety disorders in PD have not been investigated in randomized controlled trials, as stated in a review from 2011 [10]. This high comorbidity of depressive and anxiety symptoms in patients with PD a combination of anti-Parkinson and antidepressant drugs is often clinically indicated. This case demonstrates that drug-drug interactions of antidepressant and anti-Parkinson medications, particularly when including monoamine oxidase (MAO) inhibitors, may result in serotonergic overstimulation and thus require careful consideration and monitoring. Clinically, the combination of moclobemide and rasagiline is hardly encountered and moreover explicitly contraindicated by regulatory agencies. Currently, two isoforms of MAOs, MAO-A and MAO-B, have been characterized in cerebral as well as extracerebral tissues [11] by means of their expression, molecular characteristics, differences in their preferred endogenous, and exogenous substrates and inhibitor-sensitivity [12, 13]. Substrates of MAO-A include serotonin, norepinephrine, and dopamine, whereas MAO-B preferentially degrades the monoamine alkaloid phenylethylamine (regulating the release of norepinephrine and dopamine) as well as the precursor molecule of various organic compounds such as benzylamine [14, 15]. Interestingly, MAO-B activity distinctively increases with age and has repeatedly been shown to be associated with neurodegenerative processes in dementia and PD [16] strengthening the therapeutic rationale and relevance of MAO-inhibitors in PD. The antidepressant moclobemide acts as a reversible and selective inhibitor of MAO-A and is effective in treating major depression [17]. The drug is a prototype of reversible inhibitors of MAO-A agents which target this enzyme in intraneuronal presynaptic regions [18]. A near-maximum inhibitory effect in healthy male volunteers was achieved with a single dosage of 300 mg. Maximum effects, as measured by decreased plasma concentrations of the serotonin-metabolite 5-hydroxyindoleacetic acid (5-HIAA) and 3,4-dihydroxphenyl-glycol, a peripheral noradrenaline-metabolite, were apparent at moclobemide plasma levels greater than 1000 ng/ml [19]. Only one study showed the antidepressant potency of moclobemide in depressed patients with PD and the superior effectiveness of the combination therapy with the MAO-B-inhibitor selegiline on affective symptoms as well as cognition. However, the small number of included patients does not allow to draw conclusions for clinical considerations [20]. Rasagiline belongs to selective blockers of MAO-B, such as selegiline. Rasagiline shows neuroprotective actions in vitro and is used as an anti-Parkinson drug. Chronic administration of a MAO-B-selective dose (0.2 mg/kg daily for 3 weeks) increased striatal dopamine and serotonin levels, while decreasing their metabolism. The drug restored the reduced activity in aged animals in behavioral paradigms to levels seen in young animals [21, 22]. However, in a therapeutic dose alone, it did not alter cerebral monoamine levels in another animal study [22]. Pramipexole is a non-ergoline dopamine receptor agonist (D_2_-like, D_2S_ full, and D_2L_, D_3_, and D_4_ partial) for the treatment of PD and restless legs syndrome [23, 24]. Moreover, potential effects in treating symptoms of major depression have been reported [25]. Pramipexole also influences serotonergic neurotransmission by increasing activity of serotonergic neurons in the dorsal raphe. [26, 27]. Agomelatine belongs to a new generation of antidepressants since it elicits a dual mechanism of action by targeting the melatonergic system as an antagonist but also works as a serotonin 2C (5-HT_2C_) receptor antagonist. Studies have shown that treatment with agomelatine results in improvements in depressive symptoms, anxiety, and hypochondria in depressed patients [28].

## 2. Case Presentation

The 62-year-old female patient was referred to and admitted at the clinic of the Max-Planck-Institute of Psychiatry in Munich, Germany, for diagnostic clarification and treatment of recurring paroxysmal psychovegetative episodes. The patient, a retired school teacher from an urban upper-middle-class socioeconomic background, reported a history of insomnia, fatigue, and depressed mood, which preceded the onset of PD symptoms and worsened after being diagnosed with PD. She was premorbidly well-adjusted before the onset of motor symptoms and had no prior neurologic or psychiatric history. No developmental difficulties were reported. The patient's medical history includes the diagnosis of idiopathic Parkinson's syndrome, a moderate obstructive sleep apnea syndrome treated with a continuous positive airway pressure (CPAP) device, and chronic hypothyroidism following Hashimoto's disease approximately three decades ago and treated with thyroid hormone replacement since. About 6 months before clinical admission in our psychiatric ward, the patient noticed a reduced resilience to physical activity. In the last 4 months, she experienced shortness of breath after walking short distances and her general condition was affected by a feeling of physical weakness, shivering, headaches, and the sensation of facial heat. Unpredictable episodes of anxiety and vegetative symptoms occurred even out of quiescent states. Three months prior to this admission, she was assessed at an emergency department for thoracic pain and dyspnoea. Electrocardiogram (ECG), echocardiography, and blood work did not reveal any abnormalities. She was discharged with the suspected diagnosis of arterial hypertension and was started on ranolazine. Despite this intervention, her symptoms reoccurred and additionally worsened in the weeks prior to this admission. Her general practitioner (GP) recommended a beta blocker to be taken as needed. As ranolazine had no effect on her symptoms, the patient took ranolazine occasionally and discontinued the beta blocker treatment. There was no history of fever, altered sensorium, or neurological deficits except fluctuating motor symptoms associated with her diagnosis of PD. The patient described weight gain of approximately 4 kg during the last year. The patient's prevailing difficulties over the 4 months prior to admission were described as recurring paroxysmal episodes of jitteriness, perioral and acral formications, palpitations, thoracic tightness, elevated blood pressure, and a darkish red discoloration of scalp and neck. These episodes emerged unexpectedly and were not associated with physical or emotional stress or other triggering factors. The patient reported that she had contacted an emergency physician on several occasions and had been admitted to hospitals twice approximately 3-4 months before her neurologist referred her to the clinic at the Max-Planck-Institute of Psychiatry in Munich, Germany, with a working diagnosis of panic disorder. Medication at admission consisted of moclobemide (450 mg/day), agomelatine (50 mg/day), pramipexole (0.525 mg/day), rasagiline (1 mg/day), L-DOPA+benserazide retard (200+50 mg/day), ranolazine (375 mg/day), and L-thyroxine (100 *μ*g/day). The dose of moclobemide had been increased from 150 mg/day to the current dose about 1 year prior to admission. At the time of the initial mental status examination, the patient was fully alert, attentive, and oriented. She maintained eye contact and provided an informative report. No apparent abnormalities in thought form and content were observed. Cognitive and amnestic functions were intact. Her effect initially appeared euthymic with a normal range though intermittently depressed during the conversation. Her impetus towards social activities and daily activities was decreased. Her psychomotor domain was calm; her voice was quiet and showed tendencies towards decreased prosody. Both her mimic and overall expressive gestures were scarce. Severe insomnia with sleep-onset and disturbed sleep, and daytime fatigue were reported. Appetite was reported as increased over the past months. The patient did not endorse symptoms indicating specific phobia, social anxiety, or generalized anxiety. She reported paroxysmal episodes of psychovegetative strain. The patient was not suicidal; there were no safety concerns with respect to herself or others. General and neurological examination of the 62-year-old female patient (height: 165 cm, weight: 65 kg; BMI: 23.9 kg/m^2^) revealed a moderate rigor of the right upper extremity, adiadochokinesia, normal gait pattern, onychomycosis of toe nails, bilateral hallux valgus, and hyperkyphosis of the thoracic spine. No other physical or neurological abnormalities were detected. There was no postural imbalance or tremor. Vital signs at admission included blood pressure of 140/80 mmHg, heartrate of 84/min, and temperature of 36.4°C. Differential diagnoses considered at admission for reported paroxysmal episodes of psychovegetative symptoms included panic attacks/panic disorder, somatoform autonomic disorder, arterial hypertension with hypertensive exacerbations, iatrogenic hyperthyroidism, asthmatic disorder, and drug-induced serotonin toxicity. Clinical laboratory analyses did not reveal abnormal findings in CBC (complete blood count), hepatic and renal function, glucose and lipid profile, and electrolytes. Free T3 (triiodothyronine), free T4 (thyroxine), and TSH (thyroid stimulating hormone) were within normal limits. The plasma concentration of moclobemide (3310 ng/ml) was above the reference range of 300-1000 ng/ml. Plasma concentrations of agomelatine were not detectable (<1 ng/ml). We suspected potential pharmacological interactions to be a contributing factor to the symptoms reported by the patient at admission, and moclobemide, rasagiline, and ranolazine were discontinued. On the following day, the patient already experienced a decrease of psychovegetative symptoms. The blood work showed normal thyroid parameters, and no remarkable structural abnormalities of the thyroid gland were detected by sonography. Thus, we could rule out the possibility of iatrogenic hyperthyroidism. Moreover, repeated ECG examinations did not show signs of cardiac abnormalities explaining the patient's vegetative symptoms (ECG showed normal sinus rhythm, heart rate 81/min, QTc 429 ms, and vertical position). The changes of anti-parkinson medications during the stay were performed under repeated supervision of the consultant neurologists. As the discontinuation of ranolazine, which was prescribed as an antianginal medication, did not result in changes of blood pressure, we could also rule out arterial hypertension as a causative factor. The electroencephalogram (EEG) showed a regular, well-modulated, indistinct alpha-EEG with occipital accentuation and a frequency of 10-11 Hz and amplitudes reaching up to 100 *μ*V, a well-pronounced visual blockade and intermitted alpha-disintegration. A second EEG did not show any relevant changes. The magnetic resonance imaging (MRI) of the brain showed a slight expansion of the external frontal cerebrospinal fluid space and around the upper cerebellar vermis space. Disseminated, age-inappropriate, supratentorial small hyperintensities of the medullary layer were found. MRI-scan of the cervical and thoracic spine did not show any pathological findings. Due to the atypical localization of some lesions (i.e., near the corpus callosum and the right temporo-polar region), additional brain MRI-scan with contrast (gadolinium) was performed but results did not indicate an inflammatory process. In addition, lumbar puncture was discussed with the patient, who decided not to undergo this procedure after risks and benefits were explained. As the patient reported insomnia for nearly one decade and as agomelatine 50 mg daily was not effective, this medication was discontinued. After moclobemide, rasagiline, and ranolazine were discontinued based on the hypothesis of serotonergic overstimulation, no further episodic psychovegetative or panic-related symptoms occurred during hospitalization. The patient's blood pressure, which initially showed hypertensive episodes, went back to normal, and no antihypertensive medication was required. For persistent insomnia treatment trials with mirtazapine and trazodone were conducted, mirtazapine was not tolerated and trazodone was inefficient. Finally, sleep-associated symptoms slightly decreased with trimipramine 10 mg at bedtime. We established an antidepressive treatment with escitalopram, which was titrated to a dose of 10 mg. As per suggestions of consulting neurologists, pramipexole was reduced and discontinued. Because this was followed by an increase in PD-associated motor symptoms, we initiated treatment with rotigotine transdermal application of 8 mg/day. Levodopa/benserazide retard was switched to nonretard formulation of 125 mg three times a day. With this medication regimen, a stable and sufficient mobility could be achieved. Medication at discharge included escitalopram 10 mg/day, levodopa/benserazide 125 mg three times per day, rotigotine transdermal 8 mg/day, trimipramine 10 mg/day, and levothyroxine 100 *μ*g/day. The patient rated her mood as 9-10/10 on a scale from 0 to 10 with 10 being euthymic mood. The symptoms leading to admission subsided following discontinuation of MAO inhibitors moclobemide and rasagiline and did not reoccur during hospitalization.

## 3. Discussion and Conclusions

In this case report, we describe a probable drug-induced serotonergic overstimulation with paroxysmal exacerbations in a patient with PD. As other differential diagnoses could be ruled out, we suggest an interaction of several serotonergic medications, i.e., MAO-A inhibitor moclobemide and MAO-B inhibitor rasagiline as a causal factor. The hypothesis of a drug-induced symptomatology rather than genuine panic attacks is supported by subsiding of symptoms after discontinuation of both moclobemide and rasagiline, together with ranolazine. In addition, the paroxysmal episodes including jitteriness, perioral and acral formications, palpitations, thoracic tightness, elevated blood pressure, and darkish red discoloration (flush) of the scalp and neck were suggestive of drug-induced monoaminergic overstimulation. We are not aware of another case report describing serotonin toxicity with this combination in the context of Parkinson's disease. Several subtypes of panic attacks have been described in the literature [29] postulating cardiac, cardiovascular, neurological, respiratory, and vestibular classifications of panic attacks [30]. Patients presenting with chest pain frequently show symptoms/criteria for panic disorder [31] which results in diagnostic uncertainty, and in the presented case, coronary vasospasms of unknown origin had been previously suspected and treated with ranolazine. However, as the patient did not experience a significant effect on her symptoms, she reported at admission that she had been taking ranolazine not regularly and the discontinuation of ranolazine at admission did not result in the occurrence of cardiovascular symptoms. On the contrary, the patient's physical activity, i.e., walking longer distances, was restored after motor symptoms were additionally stabilized by adjusting anti-Parkinson medication as described in detail above. The serotonin syndrome is commonly perceived as a potentially fatal entity following exposure to serotonergic substances. The risk is increased with combination of two or more drugs which directly enhance postsynaptic serotonin levels. However, serotonin toxicity could reflect a continuous, dose-related phenomenon with serotonin syndrome being the maximum clinical manifestation of serotonin toxicity [32]. Frequently, the clinical symptoms of serotonin toxicity develop rapidly when a serotonergic drug is added to a preexisting medication with stimulating effects on serotonin neurotransmission [33]. It is of note that the combined use of moclobemide and rasagiline is contraindicated in the product monographs of these compounds as well as that of levodopa/benserazide and to our knowledge clinical guidelines are not supporting this combination. Due to the high prevalence of psychiatric symptoms and psychiatric comorbidities in patients with PD, combined pharmacological treatment with anti-Parkinson drugs and drugs for treatment of depression and anxiety symptoms are often inevitable. In general, hypotheses and assumptions described in this case report have certain limitations. Quantitative measurements of plasma serotonin and other monoamines as well as their respective metabolites, i.e., urinary or CSF levels of 5-HIAA, have not been determined. Thus, our conclusions with respect to the aetiology of the patient's symptoms are mainly supported by the patient's medical history, clinical symptoms, considerations of drug-drug interactions, and rapid clinical improvement after discontinuation of suspected drugs, i.e., moclobemide and rasagiline. The pharmacological interactions between moclobemide (MAO-A inhibitor) and rasagiline (MAO-B inhibitor) likely resulted in intolerable side effects as described above. We speculate that the dual inhibition of MAO-A and MAO-B may have led to MAO-inhibition comparable to the effect of irreversible MAO-inhibitors such as tranylcypromine despite the reversible nature of MAO-A inhibition by moclobemide. This may have been intermittently potentiated by dietary amines since the patient was not on a low tyramine diet [17]. It is of note that the combined use of moclobemide and rasagiline is contraindicated in the product monographs and to our knowledge clinical guidelines are not supporting this combination. We assume that moclobemide had been taken into consideration despite strong arguments against, including formal contraindication label, after a number of antidepressants were previously prescribed but eventually discontinued due to side effects and recurrence of depressive symptoms. Interestingly, when moclobemide had been given at a low dose of 150 mg daily in combination with rasagiline, the patient did indeed benefit for approximately 3 years describing improvement of depressive symptoms and good tolerability. Reported psychovegetative symptoms occurred for the first time 6 months after the dose increase of moclobemide to 450 mg daily. This long delay in onset of adverse effects related to increasing oscillations of serotonin toxicity may explain why drug-drug interactions were discarded as a potential cause by physicians. The aspect that MAO-B activity increases with age [16] may have additionally contributed to this delayed onset of symptoms. The significant reduction of MAO-B as demonstrated by Bitsios et al. who found an approximately 29% reduction in human platelet MAO-B activity after a single dosage of 450 mg moclobemide [34] and the supratherapeutic plasma concentration of moclobemide (3310 ng/ml) we detected in our patient likely constitute additional contributing factors. Hence, the following clinically relevant main aspects can be derived from the case report at hand to improve patient safety and care:

1. The importance of assessing and considering drug-drug interactions: our patient has been prescribed with a contraindicated combination of two drugs. We believe that the association of reported symptoms with adverse effects due to a drug-drug interaction was not addressed by the patient's initial prescriber, GP and ER (emergency room) physicians. The fact that the treatment was well tolerated by the patient for a prolonged period of time has likely contributed to this outcome. The case presented here highlights the importance of comprehensive and thorough assessment of pharmacological treatment and medication history and that drug-drug interactions should always be considered a potential etiological factor of clinical symptoms. This is of particular relevance for drugs where interactions have been reported as well as drugs that are not commonly prescribed or usually prescribed by different disciplines.
2. Drug-induced serotonergic overstimulation occurring on a continuous spectrum of changes in serotonergic neurotransmission: to the best of our knowledge, this is the first case report of a patient with PD with potentially drug-induced chronic serotonergic overstimulation with intermittent clinical exacerbations mimicking panic attacks. Most likely, this was caused by combined treatment with an antidepressant (moclobemide; MAO-A inhibitor) and anti-Parkinson medication (rasagiline; MAO-B inhibitor). Interestingly, signs of serotonin toxicity probably developed following an increase of moclobemide from 150 to 450 mg daily and “although polypharmacy is an important etiological factor in the development of serotonin syndrome per se, dose and speed of distribution may determine its severity" [35]. We believe that this case report can support the clinically relevant assumption that drug-induced serotonergic overstimulation may occur on a continuous spectrum of changes in serotonergic neurotransmission which may present as mild clinical symptoms, clinical exacerbations, or the potentially life-threatening condition of serotonin syndrome.

## Abbreviations

5-HIAA: 5-Hydroxyindoleacetic acid
5-HT: 5-Hydroxytryptamine/serotonin
CBC: Complete blood count
ECG: Electrocardiogram
EEG: Electroencephalogram
ER: Emergency room
L-DOPA: Levodopa/L-3,4-dihydroxyphenylalanine
GP: General practitioner
MAO: Monoamine oxidase
MRI: Magnetic resonance imaging
PD: Parkinson's disease
REM: Rapid eye movement
T3: Triiodothyronine
T4: Thyroxine
TSH: Thyroid stimulating hormone.
